# Determinants and impact of multidrug antibiotic resistance in pathogens causing ventilator-associated-pneumonia

**DOI:** 10.1186/cc7119

**Published:** 2008-11-17

**Authors:** Pieter O Depuydt, Dominique M Vandijck, Maarten A Bekaert, Johan M Decruyenaere, Stijn I Blot, Dirk P Vogelaers, Dominique D Benoit

**Affiliations:** 1 Department of Intensive Care, Ghent University Hospital, De Pintelaan 185, B-9000 Gent, Belgium; 2 Department of Applied Mathematics and Computer Science, Ghent University, Krijgslaan 281 S9, B-9000 Gent, Belgium; 3 Department of Internal Medicine and Infectious Diseases, Ghent University Hospital, De Pintelaan 185, B-9000 Gent, Belgium

## Abstract

**Introduction:**

The idea that multidrug resistance (MDR) to antibiotics in pathogens causing ventilator-associated pneumonia (VAP) is an independent risk factor for adverse outcome is still debated. We aimed to identify the determinants of MDR versus non-MDR microbial aetiology in VAP and assessed whether MDR versus non-MDR VAP was independently associated with increased 30-day mortality.

**Methods:**

We performed a retrospective analysis of a prospectively registered cohort of adult patients with microbiologically confirmed VAP, diagnosed at a university hospital intensive care unit during a three-year period. Determinants of MDR as compared with non-MDR microbial aetiology and impact of MDR versus non-MDR aetiology on mortality were investigated using multivariate logistic and competing risk regression analysis.

**Results:**

MDR pathogens were involved in 52 of 192 episodes of VAP (27%): methicillin-resistant *Staphylococcus aureus *in 12 (6%), extended-spectrum β-lactamase producing *Enterobacteriaceae *in 28 (15%), MDR *Pseudomonas aeruginosa *and other non-fermenting pathogens in 12 (6%). Multivariable logistic regression identified the Charlson index of comorbidity (odds ratio (OR) = 1.38, 95% confidence interval (CI) = 1.08 to 1.75, p = 0.01) and previous exposure to more than two different antibiotic classes (OR = 5.11, 95% CI = 1.38 to 18.89, p = 0.01) as predictors of MDR aetiology. Thirty-day mortality after VAP diagnosis caused by MDR versus non-MDR was 37% and 20% (p = 0.02), respectively. A multivariate competing risk regression analysis showed that renal replacement therapy before VAP (standardised hazard ratio (SHR) = 2.69, 95% CI = 1.47 to 4.94, p = 0.01), the Charlson index of comorbidity (SHR = 1.21, 95% CI = 1.03 to 1.41, p = 0.03) and septic shock on admission to the intensive care unit (SHR = 1.86, 95% CI = 1.03 to 3.35, p = 0.03), but not MDR aetiology of VAP, were independent predictors of mortality.

**Conclusions:**

The risk of MDR pathogens causing VAP was mainly determined by comorbidity and prior exposure to more than two antibiotics. The increased mortality of VAP caused by MDR as compared with non-MDR pathogens was explained by more severe comorbidity and organ failure before VAP.

## Introduction

Ventilator-associated pneumonia (VAP) is a major infectious complication in critically ill patients in terms of its incidence and associated mortality and morbidity [1-4]. A clinical suspicion of VAP is responsible for the majority of antibiotic prescription in the intensive care unit (ICU) [5]. Estimates of attributable mortality of VAP range from 0% to as high as 50% [6], and this variability is thought to depend on several factors such as admission diagnosis of patients in the study, severity of illness at the time of VAP, type of microbial pathogen and whether appropriate antibiotic treatment is provided in a timely manner [7-10].

Microbial pathogens involved in VAP are frequently multidrug resistant (MDR), which challenges the appropriateness of empirical antibiotic prescription [10]. Furthermore, MDR inflates antibiotic consumption because it necessitates empirical use of broad-spectrum antibiotics, often in combination therapy [1], and it hampers subsequent de-escalation of this therapy [11]. Several authors have observed increased mortality in VAP caused by MDR pathogens as compared with other bacterial pathogens, which they have attributed to a higher risk of initial inappropriate antibiotic therapy in these patients [10-13] or to increased intrinsic virulence of the pathogen [14,15]. Others have taken the alternative view that increased mortality in MDR VAP is largely due to confounding [16-19] by the preferential occurrence of MDR infection in a subset of ICU patients with *a priori *decreased odds for survival, that is, those patients with a prolonged duration of mechanical ventilation and previous antibiotic treatment [20].

In the present study, we aimed to identify the risk factors for MDR as compared with non-MDR microbial aetiology of VAP, and tested the hypothesis that MDR, as compared with non-MDR, aetiology of VAP is an independent predictor of mortality using a multivariate competing risk analysis according to the methodology of Fine and Gray [21].

## Materials and methods

### Study design, patients and clinical setting

To examine the determinants of mortality, a prospective cohort study was performed recruiting all patients with microbiologically confirmed VAP during a three-year period (1 April 2004 to 31 March 2007) in our 54-bed medical and surgical ICU of the 1060-bed Ghent University Hospital. To address the determinants for MDR bacterial aetiology of VAP, we performed a subsequent case-control study of patients with MDR VAP. Patients with VAP caused by non-MDR pathogens acted as controls. All patients aged 16 years and older and ventilated for at least 48 hours were assessed daily for evidence of VAP. Patients who were chronically mechanically ventilated were excluded. Only microbiologically confirmed episodes of VAP were considered for analysis. The study was approved by the Ethics Committee of Ghent University Hospital. Written informed consent to obtain patients' data was given by the patient or the patient's representative if the patient was unable to give consent.

Routine microbiological work-up of airway samples consisted of rapid Gram-staining and semi-quantitative culturing of tracheal aspirate. On clinical request (in cases of discrepancy between likely clinical diagnosis of VAP and semi-quantitative microbiological results), quantitative culturing was performed on tracheal aspirate or on broncho-alveolar lavage fluid obtained by means of fibre-optic bronchoscopy. Plate quantitation for semiquantitative cultures and use of selective media to identify MDR pathogens was performed as described previously [22,23]. Semiquantitative scoring was derived from streaking and diluting the specimen in three segments, scored as few (+-) for less than 10 colonies, light (+), moderate(++) and heavy (+++) growth when moderate to heavy growth was observed in first, second and third streaks respectively. Antibiotic susceptibility was determined according to methods recommended by the Clinical and Laboratory Standards Institute [24].

At our hospital, initial antibiotic therapy in ICU-acquired infection is guided by surveillance cultures, as described previously [22,23,25]. At clinical diagnosis of VAP, the following antibiotics were prescribed if surveillance cultures did not grow *Pseudomonas aeruginosa *or MDR organisms: a second-generation cephalosporin or amoxicillin-clavulanic acid in pneumonia diagnosed within one week or less after ICU admission of a patient without prior antibiotic exposure; or an antipseudomonal β-lactam in patients with prior antibiotic exposure or an ICU stay of more than one week. If additional risk factors for *P. aeruginosa *were present (e.g. bronchiectasis, corticosteroid therapy) or if *P. aeruginosa *was isolated from surveillance cultures, an antipseudomonal β-lactam treatment was complemented with an aminoglycoside or fluoroquinolone. In patients with surveillance cultures growing MDR organisms, initial antibiotic therapy, consisting of an antipseudomonal β-lactam antibiotic or carbapenem was complemented by a glycopeptide, fluoroquinolone or aminoglycoside as appropriate; alternatively, targeted therapy directed at the MDR pathogen was provided.

### Data collected

Data collected at ICU admission included demographics, admission diagnosis, presence of comorbidity, severity of illness on admission as assessed by Acute Physiology and Chronic Health Evaluation (APACHE) II score, presence of coma (defined by Glasgow Coma Scale (< 6) and development of circulatory shock on ICU admission (defined as requirement of vasopressor therapy after restoring intravascular volume within 48 hours of ICU admission). Presence of comorbidity was quantified using the Charlson index of comorbidity [26], as described previously [24,27].

Data collected at diagnosis of VAP were prior duration of mechanical ventilation (days), prior antibiotic therapy within the same hospitalisation period, number of infectious episodes and number of different antibiotic classes prescribed. We recorded a diagnosis of underlying Acute Respiratory Distress Syndrome (ARDS) and underlying acute kidney injury requiring renal replacement therapy, if present at least two days (day -2) before VAP, and the presence or absence of shock on ICU admission. Clinical pulmonary infection score (CPIS) was calculated at suspicion of VAP to corroborate clinical diagnosis [28]. Microbial aetiology was recorded if available (see definitions below). Sequential Organ Failure Assessment (SOFA) score was calculated on the day of diagnosis of VAP, as well as at day -2 and two days after (day +2) diagnosis of VAP. The SOFA score is a scoring system quantifying the extent of a critically ill patient's organ dysfunction or failure, and is composed of six subscores, one each for the respiratory, cardiovascular, hepatic, coagulation, renal and neurological systems [29].

Antibiotic prescription on diagnosis of VAP was noted: antibiotic prescription on the same calendar day and the calendar day after clinical diagnosis of VAP was considered as antibiotic therapy within 24 hours and 48 hours of VAP, respectively. Primary outcome parameter was 30-day mortality after diagnosis of VAP.

### Definitions

VAP was considered clinically likely if a new or progressive and persistent infiltrate was present on chest X-ray together with at least two signs of systemic inflammation, such as fever with a temperature higher than 38°C or hypothermia with a temperature lower than 36°C, leucocytosis (>11,000 white blood cell count (WBC)/mm^3^) or leucopenia (<4000 WBC/mm^3^), rising C-reactive protein (> 2 mg/dL within 48 hours), and with at least one sign of local inflammation such as purulent sputum and a decrease of partial pressure of oxygen in arterial blood (PaO_2)_/fraction of inspired oxygen (FiO_2_) of at least 10%. Moreover, a CPIS of at least six was required to maintain diagnosis of clinically likely VAP [28].

Clinically likely VAP was considered as microbiologically confirmed if: a pathogen showed ++ or +++ semiquantitative culture or more than 10^5 ^colony forming units (CFU)/mL quantitative growth on a good quality endotracheal aspirate; growth of ++ semiquantitative culture of more than 10^4 ^CFU/mL on broncho-alveolar lavage fluid; growth of at least + together with positive Gram-staining if antibiotic therapy had been initiated or changed within 48 hours before sampling; or when a pathogen was isolated both from endotracheal aspirate and blood cultures. Based on a previous in-house analysis where semiquantitative and quantitative cultures on endotracheal aspirate correlated well (data not shown), and supported by other reports, we rely both on semiquantitative and quantitative cultures for microbiological confirmation of VAP [30,31]. If more than one pathogen grew above the semiquantitative or quantitative threshold, VAP was considered polymicrobial and if at least one MDR pathogen grew above these thresholds, VAP was considered as MDR.

The following pathogens were considered as MDR: methicillin-resistant *Staphylococcus aureus *(MRSA), extended-spectrum β-lactamase producing Gram-negative *Enterobacteriaceae *(ESBL), *Pseudomonas aeruginosa *and other non-fermenting organisms (*Acinetobacter baumannii*, *Stenotrophomonas maltophilia*) resistant for three or more of the following antibiotic classes: antipseudomonal cephalosporins or penicillins, carbapenems, fluoroquinolones and aminoglycosides (MDR NF). Antimicrobial therapy within 24 hours and 48 hours of diagnosis of VAP was considered appropriate if it included at least one antimicrobial drug with *in vitro *activity against the aetiologic agent identified. ARDS was defined according to the criteria of the American-European consensus conference [32], and shock was defined as the requirement of vasopressor therapy (noradrenaline or adrenaline) to restore adequate arterial pressure and organ perfusion despite appropriate intravenous fluid substitution.

### Statistics

Continuous variables are described as mean (± standard deviation), median (25th to 75th percentile) and categorical variables are described as n (%). For comparative tests on continuous variables, the Mann-Whitney U test and student's t-test were used as appropriate, depending on variable distribution. For categorical variables, the Pearson chi-square test or the Fisher's exact test were used as appropriate. The response variable used in the mortality analyses was vital status (alive or dead) 30 days after diagnosis of VAP. In patients with multiple episodes of VAP, only the first microbiologically confirmed VAP was retained for further analysis. Logistic regression analysis was used to assess the multivariate relation between multiple patient characteristics and the probability of involvement of MDR as compared with non-MDR pathogens in VAP. To adjust for the association of MDR versus non-MDR microbial aetiology on 30-day mortality after diagnosis of VAP on potential confounders and to check whether MDR is a independent predictor, we performed a competing risk analysis using the Fine and Gray model [21], with 30-day mortality after diagnosis of VAP as the endpoint of interest, and discharge alive from the hospital within 30 days after diagnosis of VAP as the competing risk.

Recently some authors discussed the application of these recently developed models in the specific ICU-setting where censoring due to discharge alive from the ICU violates the assumption of non-informative censoring [33,34]. Consequently, standard survival methods which rely on non-informative censoring appear not to be appropriate here [35,36]. As the primary aim was to determine whether MDR constituted an independent risk factor for mortality in the presence of other covariates, the enter method was primarily used, complemented with stepwise forward and backward analysis to test stability of the models. Overall, predictors showing a p < 0.1 association with in-hospital mortality in univariate analysis as well as those variables that seemed clinically important were incorporated in the regression analyses. Correlation matrixes for all predictors included in the regression analyses were constructed to avoid inclusion of significantly associated sets of predictors and to limit the risk of colinearity.

When appropriate, odds ratios (OR) and 95% confidence intervals (CI) were reported, and the Hosmer-Lemeshow goodness-of-fit test and the area under the curve of the resulting receiver operator curve were provided. Results from the competing risk analysis were reported as sub-hazard ratios, which are the ratios of hazards associated with the cumulative incidence function. The various models were tested for the presence of clinically significant interaction. Statistical analyses were executed with SPSS 11.0 (SPSS Inc. Chicago, IL) and the R 2.6.2 software package [37]. The competing risk analysis was performed using the *crr *routine available in the *cmprsk *package developed by Gray [38]. All tests used were two-tailed and statistical significance was defined as p < 0.05.

## Results

**Figure 1:**
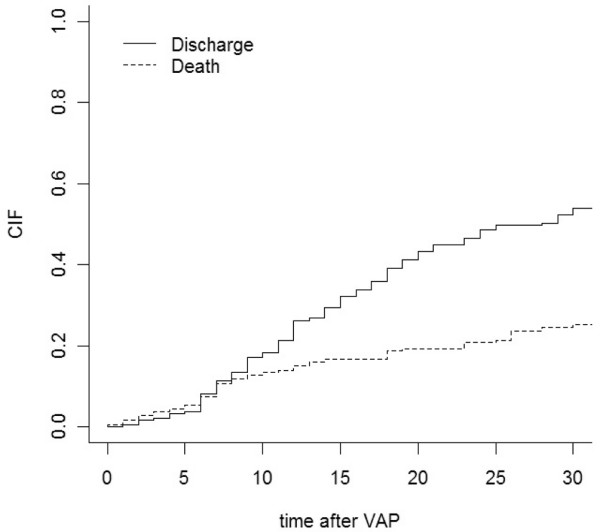
Cumulative incidence function of death 30 days after diagnosis and of being discharged alive.

During the study period, microbiologically confirmed VAP was diagnosed in 192 patients. MDR pathogens were isolated in 52 of 192 (27%) first episodes and in 11 of 34 (32%) subsequent episodes of VAP. Systematic oral, nasal, urinary and rectal surveillance cultures obtained within the first 48 hours of ICU admission revealed presence of MDR pathogens in seven patients (4%). Of the 192 patients included in the study, 47 patients (24.5%) died within 30 days of VAP diagnosis. The estimated cumulative incidence function of death was 16.6% on day 10 and 24.5% on day 30. For the competing risk (discharged alive from the hospital) the estimated cumulative incidence function was 32% and 54%, respectively (Figure 1).

### Risk factors for involvement of MDR pathogens in VAP

**Figure 2:**
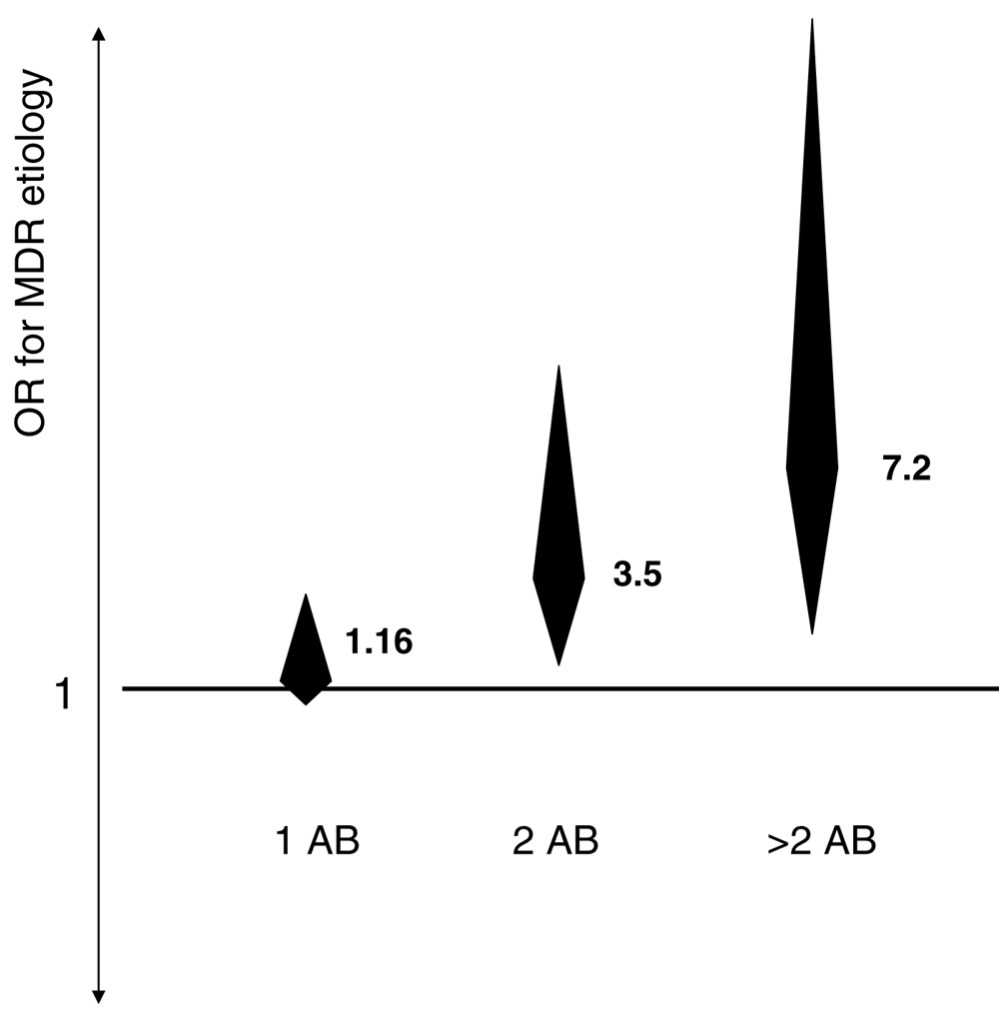
**Odds ratio for risk of multidrug-resistant microbial aetiology of ventilator-associated pneumonia**. Odds ratio for risk of multidrug-resistant (MDR) microbial aetiology of ventilator-associated pneumonia (VAP) with increasing previous antibiotic exposure, expressed as the number of antibiotic classes* received before VAP diagnosis. * β-lactam antibiotics (penicillins and cephalosporins), carbapenems, fluoroquinolones, aminoglycosides, glycopeptides and linezolid, other antibiotics such as cotrimoxazole and colistin.

Characteristics of patients with VAP caused by MDR versus non-MDR pathogens are provided in table 1, as well as the predominant pathogen identified. The univariate odds ratio for isolating a MDR pathogen in patients previously exposed to an increasing number of antimicrobial classes is shown in figure 2. Rates of appropriate antibiotic therapy achieved within 24 hours and 48 hours after diagnosis of VAP were lower in patients with MDR as compared with other pathogens, and 30-day, ICU- and in-hospital mortality were significantly higher in patients with VAP caused by MDR than in patients with non-MDR VAP.

**Table 1:** Characteristics of patients with ventilator-associated pneumonia caused by multidrug resistant (n = 52) and non-MDR (n = 140) pathogens*

Characteristics	MDR (n = 52)	Non-MDR (n = 140)	p value
**Demographics**			
Age, years	63 ± 13	58 ± 17	0.07
Male gender	41 (79%)	97 (69%)	0.37
**Characteristics before VAP**			
Charlson index	2 (1 to 3)	1 (0 to 3)	< 0.001
APACHE II upon ICU admission	22 ± 9	20 ± 9	0.37
Coma on ICU admission	6 (12%)	54 (39%)	< 0.001
Shock on ICU admission	20 (38%)	56(40%)	0.87
ARDS before VAP	25 (48%)	29 (21%)	0.001
RRT before VAP	14 (27%)	18 (13%)	0.03
Duration of ventilation before VAP, days	9 (5 to 19)	7 (4 to 13)	0.008
Duration hospitalisation before VAP, days	0 (0 to 6)	0 (0 to 1)	0.001
Antibiotic exposure before VAP			
No antibiotics	6 (11%)	32 (23%)	0.11
1 antibiotic class	15 (29%)	73 (52%)	0.005
2 antibiotic classes	13 (25%)	24 (17%)	0.22
> 2 antibiotic classes	18 (35%)	11 (8%)	< 0.001
SOFA 2 days before VAP	5 (3 to 8)	5 (3 to 8)	0.83
**Characteristics of VAP**			
SOFA at VAP	7 (3 to 10)	6 (4 to 9)	0.27
Delta SOFA^□^	0.6 ± 3.3	0.6 ± 3.5	0.88
VAP with shock	15 (29%)	26 (19%)	0.16
Gram-negative aetiology	40 (77%)	122 (87%)	0.39
*Enterobacteriaceae*	28 (54%)	66 (46%)	0.07
*Pseudomonas aeruginosa*	11 (21%)	40 (29%)	0.98
Non-fermenter other than *P. aeruginosa*	1 (2%)	4 (3%)	0.32
Other Gram-negative^†^	0	12 (9%)	0.001
Gram-positive aetiology	12 (23%)	18 (13%)	0.39
*Staphylococcus aureus*	12 (23%)	13 (9%)	0.14
*Streptococcus pneumoniae*	0	5 (3%)	0.01
Appropriate therapy < 24 hours	41 (79%)	122 (87%)	0.16
Appropriate therapy < 48 hours	45 (87%)	133 (95%)	0.05
Subsequent VAP	10 (19%)	24 (17%)	0.83
**Outcome parameters:**			
ICU-length of stay following VAP, days	18 (8 to 37)	15 (8 to 29)	0.34
Died 30 days after VAP diagnosis	19 (37%)	28 (20%)	0.02
Died at ICU	22 (42%)	29 (21%)	0.006
Died in hospital	28 (54%)	41 (29%)	0.001
Discharged to other hospital and lost to follow up	2	3	
Referred to chronic health care facility	0	4	
Still hospitalised at time of analysis	4	3	

*Data are presented as mean ± SD, median (25^th ^to 75th percentile) or number (%).

^□^Delta SOFA: SOFA two days after VAP minus SOFA two days before VAP.

† Other Gram-negative: *Haemophilus influenzae *in 11, *Moraxella catarrhalis *in 1.

^◇^β-lactam antibiotics (penicillins and cephalosporins), carbapenems, fluoroquinolones, aminoglycosides, glycopeptides and linezolid, other antibiotics (cotrimoxazole, colistin).

APACHE = Acute Physiology and Chronic Health Evaluation; ARDS = acute respiratory distress syndrome; ICU, Intensive Care Unit; MDR, Multidrug-resistant; RRT, Renal Replacement Therapy; SOFA = sequential organ failure assessment; VAP, Ventilator-associated Pneumonia.

Results of the multivariable analysis of predictors of MDR as compared with non-MDR microbial aetiology of VAP are shown in table 2. We included exposure to one, two and more than two antibiotic classes (with no prior antibiotics as reference category), together with duration of mechanical ventilation (days) prior to VAP as predictors, as these are known risk factors for MDR [20], as well as those predictors that showed a significant association (p < 0.1) in univariate analysis. Exposure to more than two antibiotic classes during hospitalisation before VAP diagnosis was significantly associated with MDR aetiology in enter and backward stepwise regression analysis.

**Table 2:** Multivariable regression analysis of factors associated with the involvement of MDR pathogens in VAP (n = 192).

Predictor	Parameter estimate	OR	CI	p value
**Enter method**				
Age	0.01	1.01	0.99 to 1.03	0.23
Charlson index	0.85	1.09	0.93 to 1.27	0.28
Coma on ICU admission	-0.83	0.44	0.19 to 1.03	0.06
ARDS before VAP	0.52	1.69	0.86 to 3.31	0.13
RRT before VAP	-0.42	0.66	0.31 to 1.42	0.29
Duration of hospitalisation before VAP	0.006	1.01	1.00 to 1.02	0.20
One antibiotic before VAP	0.06	1.06	0.39 to 2.92	0.90
Two antibiotic classes before VAP	0.85	2.34	0.82 to 6.71	0.11
More than two antibiotic classes before VAP	1.37	3.93	1.26 to 12.23	0.02
Constant	-2.72			
**Backward stepwise**				
Coma on ICU admission	-1.12	0.32	0.14 to 0.73	0.01
Two antibiotic classes before VAP	0.88	2.41	1.22 to 4.79	0.01
More than two antibiotic classes before VAP	1.50	4.47	2.15 to 9.31	< 0.001
Constant	-1.50			

Overall correct prediction: 77%.

Hosmer-Lemeshow goodness-of-fit: Chi-square 4.74, p = 0.8, eight degrees of freedom. ROC curve: area under the curve = 0.80 (0.73 to 0.86).

ARDS = acute respiratory distress syndrome; CI = confidence interval; ICU = intensive care unit; MDR = multidrug resistant; OR = odds ratio; RRT = renal replacement therapy; VAP = ventilator-associated pneumonia.

### Risk factors for 30 days mortality following VAP

Characteristics of nonsurvivors and survivors are shown in univariate analysis in table 3. Results of the Fine and Gray regression model are shown in table 4. MDR bacterial aetiology was not independently associated with mortality. Renal replacement therapy before diagnosis of VAP (standardised hazard ratio (SHR) = 2.69, 95% CI = 1.47 to 4.94, p = 0.001), the Charlson index of comorbidity (SHR = 1.21, 95% CI = 1.03 to 1.41, p = 0.02) and shock on ICU admission (SHR = 1.86, 95% CI = 1.03 to 3.35, p = 0.04) were significant predictors of 30-day mortality after VAP diagnosis.

**Table 3:** Variables associated with 30-day mortality after VAP diagnosis in univariate analysis (n = 192)*

Characteristics	Nonsurvivors (n = 47)	Survivors (n = 145)	p value
**Demographics**			
Age	62 ± 14	58 ± 17	0.12
Male gender	14 (30%)	40 (28%)	0.71
**Characteristics before VAP**			
Charlson index	2 (1 to 3)	1 (0 to 3)	0.01
APACHE II on ICU admission	20 ± 8	21 ± 9	0.87
Shock on ICU admission	26 (55%)	50 (35%)	0.016
ARDS	21 (45%)	33 (23%)	0.005
RRT before VAP	17 (36%)	15 (10%)	< 0.001
Duration of mechanical ventilation before VAP, days	9 (5 to 16)	7 (3 to 14)	0.14
Number of infectious episodes before VAP	1 (1 to 1)	1 (1 to 1)	0.243
SOFA 2 days before VAP	7 (5 to 9)	5 (3 to 7)	0.001
**Characteristics at diagnosis of VAP**			
SOFA at VAP	9 (5 to 11)	5 (4 to 8)	< 0.001
Delta SOFA^□^	1 (0 to 4)	0 (-1 to 1)	< 0.001
VAP with shock	19 (40%)	22(15%)	< 0.001
Microbial aetiology of VAP			
MDR aetiology	19 (40%)	33 (23%)	0.02
Gram-negative aetiology	40 (85%)	122 (84%)	0.39
*Enterobacteriaceae*	24 (62%)	70 (48%)	0.71
*Pseudomonas aeruginosa*	12 (26%)	39 (27%)	0.84
Non-fermenter other than *P. aeruginosa*	3 (6%)	2 (1%)	0.006
Other Gram-negative^†^	1(2%)	11 (8%)	0.001
Gram-positive aetiology	7(15%)	23(16%)	0.39
*Staphylococcus aureus*	7 (15%)	18 (12%)	0.37
*Streptococcus pneumoniae*	0	5 (4%)	0.01
Subsequent VAP	4 (9%)	30(21%)	0.08
**Treatment characteristics**			
Appropriate therapy < 24 hours^¶^	38 (83%)	125(86%)	0.63
Appropriate therapy < 48 hours^¶^	43 (94%)	135 (93%)	0.99

*Data are presented as mean (± SD), median (25^th ^to 75th percentile) or number (%).

^□^Delta SOFA: SOFA two days after VAP minus SOFA two days before VAP.

† Other Gram-negative: *Haemophilus. influenzae *in 11, *Moraxella catarrhalis *in 1.

^¶^Data about antibiotic therapy were available in 187 patients (50 nonsurvivors and 137 survivors).

APACHE = Acute Physiology and Chronic Health Evaluation; ARDS = acute respiratory distress syndrome; ICU, Intensive Care Unit; MDR, Multidrug-resistant; RRT, Renal Replacement Therapy; SOFA = sequential organ failure assessment; VAP, Ventilator-associated Pneumonia.

**Table 4:** Fine and Gray multivariate analysis of factors associated with 30-day mortality after VAP diagnosis (n = 192). The five variables selected on the basis of univariate analyses (n = 192).

Predictor	Parameter estimate	SHR	CI	p value
**Enter method**				
MDR aetiology	0.33	1.39	0.77 to 2.50	0.28
ARDS before VAP	0.42	1.52	0.83 to 2.79	0.18
RRT before VAP	0.79	2.20	1.18 to 4.13	0.01
Charlson index of comorbidity	0.18	1.20	1.02 to 1.40	0.04
Shock on ICU admission	0.55	1.73	0.96 to 3.12	0.07
**Forward and backward stepwise**				
RRT before VAP	0.99	2.69	1.18 to 4.28	0.001
Charlson index of comorbidity	0.19	1.21	1.03 to 1.41	0.02
Shock on ICU admission	0.62	1.73	1.03 to 3.35	0.04

ARDS = adult respiratory distress syndrome; CI = confidence interval; ICU = intensive care unit; MDR = multidrug resistant pathogen; RRT = renal replacement therapy; SHR = subdistribution hazard ratio; VAP = ventilator-associated pneumonia.

## Discussion

Only 3% of our patients were colonised with MDR pathogens on ICU admission (as detected by our routine surveillance cultures [23-25]) but MDR pathogens were involved in between one-quarter and one-third of cases of VAP. This underscores the pivotal role of the ICU as a specific nosocomial environment promoting the emergence and acquisition of MDR pathogens. A major determinant of the risk of MDR pathogens causing VAP was previous antibiotic selection pressure: exposure to more than two different classes of antibiotics since hospital admission remained strongly associated with MDR involvement after adjustment for exposure time and degree of organ failure before diagnosis of VAP. Previous antibiotic exposure has been identified as a risk factor for MDR microbial aetiology of VAP in several studies [7,20,39,40]: our study adds that this risk is especially high when a high 'burden' of this selection pressure is present (figure 2).

Coma upon ICU admission showed a protective effect on the risk of finding a MDR pathogen in VAP. This effect is likely explained by the fact that in our cohort, coma as a covariate identifies a subset which mainly consists of younger neurotrauma patients. As neurotrauma patients are at high risk for early-onset VAP, our study design, where we only included the first microbiologically confirmed episode of VAP, favoured this association.

To test the hypothesis of whether a MDR microbial aetiology of VAP was independently associated with increased 30-day mortality after diagnosis of VAP, as compared with a non-MDR bacterial cause, we performed a Fine and Gray regression analysis, with discharge from the hospital alive as a competing event to mortality. Survival analytic methods, such as Cox regression analysis, have recently been challenged for their appropriateness to evaluate ICU-mortality: the main criticism applies to the fact that in order to yield correct results, censoring must be independent of the outcome of interest (i.e. mortality) [33-36]. If censoring results from ICU- or hospital discharge, this assumption is not correct, as patients discharged alive are at much lower risk of mortality than patients remaining at the ICU. This problem is bypassed by multivariate logistic regression analysis, but here crucial information may be lost as the time to death is not taken into account. The Fine and Gray analysis, although closely related to logistic regression, extends this model by incorporating different exposure times in the ICU. Using the Fine and Gray model, the association between increased (cumulative) 30-day mortality following MDR versus a non-MDR VAP diagnosis was no longer significant after appropriate adjustment for comorbidity and some measures of more severe critical illness, such as shock on ICU admission, underlying ARDS and severe acute kidney injury requiring renal replacement therapy. As such, the isolation of MDR versus other pathogens in our study behaved as a marker of a category of patients with a lower *a priori *chance of ICU survival.

The lack of association between antimicrobial resistance and mortality has also been observed by Combes and colleagues in patients with VAP caused by *P. aeruginosa *and *S. aureus *[16,17], and by Blot and colleagues in patients with Gram-negative bacteraemia [41]. In contrast, in a retrospective study on bacteraemic VAP, MRSA and MDR *P. aeruginosa *were independently associated with increased mortality [24]. As this retrospective study consisted of more severely ill patients with a higher mortality rate, a possibility remains that the impact of MDR varies according to different categories of patients. Alternatively, residual confounding may be of concern in this study, because underlying critical illness at diagnosis of VAP was not accounted for. Part of the controversy of whether involvement of MDR in nosocomial infection is an independent risk factor for mortality possibly stems from inclusion of different sets of covariates in regression models or from different matching criteria in matched cohort studies. When assessing the impact of MDR on outcome, measures of severity of illness more close to the time of diagnosis of VAP, rather than on ICU admission, probably allow for better adjustment in multivariable regression or for better balancing patient cohorts in a matched cohort analysis [18,42]. Yet, care must be taken to assess severity of illness sufficiently before the onset of VAP, because incipient VAP itself may increase the measure of severity of illness.

Acute kidney injury requiring renal replacement therapy preceding development of VAP was an independent predictor of mortality. An excess mortality associated with the requirement for renal replacement therapy in ICU patients has been recently demonstrated in a large multicentric matched cohort analysis [43]. Early appropriate antibiotic therapy on the other hand was not associated with mortality. This lack of association is probably due to underpowering as few patients received inappropriate therapy: appropriate antibiotics were administered within 24 hours and within 48 hours in 85% and 93% of episodes, respectively, and in the subgroup of patients with septic shock, in which early appropriate antibiotic therapy may have the greatest impact [13], these figures were 90% and 100%, respectively.

Our study has several limitations. Firstly, determinants of MDR were identified using a case-control design, with patients with VAP caused by non-MDR pathogens as controls rather than patients at risk of developing VAP (source patients). As antibiotic exposure is likely to suppress the growth of susceptible bacteria, these control patients may have received fewer antibiotics than the overall source patients, leading to overestimation of the association between antibiotic exposure and MDR [44]. Other bias caused by different time-at-risk and comorbidity was reduced by our multivariable analysis, adjusting for duration of prior hospitalisation and the Charlson index of comorbidity, but bias may not have been completely eliminated. However, it is more likely that insufficient elimination of confounding would have lead to a detection of a false association than obscuring a real association. Secondly, as we defined MDR as a set of pathogens rather than antibiotic resistance in a single microbial species, our study may be underpowered to detect the possible deleterious impact of MDR in VAP caused by a single microbial species, such as *S. aureus *of *P. aeruginosa*. Similarly, elucidating the relation between patterns of antimicrobial resistance encountered in pathogens causing VAP and previous antimicrobial prescription would require a larger and preferably multicentric study. Although our current approach may have lead to missing a significant association between antibiotic resistance in a particular pathogen and outcome, at the very least this could not be detected in our three-year dataset derived from a large tertiary ICU with a resistant microbial flora. We believe therefore that even if such an association was present, the strength of such association was probably small.

Secondly, the small sample size permitted the inclusion of only a limited number of covariates in our multivariate analysis. However, as the aim of our study was primarily to test whether MDR is independently associated with mortality, finding at least one regression model where MDR was not a significant predictor was sufficient to falsify the null-hypothesis.

## Conclusion

In our cohort of patients with VAP, exposure to more than two antibiotic classes after hospital admission was identified as the most important risk factor for a MDR microbial aetiology of VAP. A MDR, as compared with a non-MDR, bacterial cause of VAP was not an independent risk factor for ICU-mortality in a setting where rates of early appropriate antibiotic therapy were high.

## Key messages

• The risk of MDR in VAP is especially high after exposure to more than two antibiotics.

• Underlying organ failure rather than MDR was predictive for outcome in VAP.

## Abbreviations

APACHE: Acute Physiology and Chronic Health Evaluation; ARDS: Acute Respiratory Distress Syndrome; CFU: colony forming units; CI: confidence interval; CPIS: Clinical Pulmonary Infections Score; ESBL: extended spectrum β-lactamase producing *Enterobacteriaceae*; ICU: intensive care unit; MDR: multidrug antbiotic resistant; MRSA: methicillin-resistant *Staphylococcus aureus*; OR: odds ratio; SHR: standardised hazard ratio; SOFA: sequential organ failure assessment; VAP: ventilator-associated pneumonia; WBC: white blood cell count.

## Competing interests

The authors declare that they have no competing interests.

## Authors' contributions

PD designed the study and drafted the manuscript. PD, MB and DB performed the statistical analysis. PD, DV and DB acquired the data. All authors participated to the analysis and interpretation of the data. DV, JD and SB critically revised the manuscript and contributed to intellectual content. All authors read and approved the final version of the manuscript.
